# Comparative Evaluation of the Nutrients, Phytochemicals, and Antioxidant Activity of Two Hempseed Oils and Their Byproducts after Cold Pressing

**DOI:** 10.3390/molecules27113431

**Published:** 2022-05-26

**Authors:** Cristina Occhiuto, Gianluigi Aliberto, Mariarosaria Ingegneri, Domenico Trombetta, Clara Circosta, Antonella Smeriglio

**Affiliations:** 1 Foundation “Prof. Antonio Imbesi”, University of Messina, Piazza Pugliatti 1, 98122 Messina, Italy; cristina.occhiuto@unime.it (C.O.); glglbr03@gmail.com (G.A.); 2 Department of Chemical, Biological, Pharmaceutical and Environmental Sciences, University of Messina, Viale Ferdinando Stagno d’Alcontres 31, 98166 Messina, Italy; mariarosaria.ingegneri@unime.it (M.I.); clara.circosta@unime.it (C.C.); antonella.smeriglio@unime.it (A.S.)

**Keywords:** *Cannabis sativa* L. (industrial hemp), USO 31, Futura 75, cold-pressed seed oil, hemp byproducts, hemp meal, nutrients, anti-nutritional factors, secondary metabolites, antioxidant properties

## Abstract

Recently, there has been a growing interest in the recovery of agri-food waste within the circular economy perspective. In this study, the nutritional, phytochemical, and biological features of the cold-pressed hempseed oil (HSO) and hempseed meal (HSM) of two industrial hemp varieties (USO 31 and Futura 75, THC ≤ 0.2%) were evaluated. The HSOs showed a high total phenols and flavonoid content, which were confirmed by LC-DAD-ESI-MS analysis, with rutin as the most abundant compound (56.93–77.89 µg/100 FW). They also proved to be a rich source of tocopherols (81.69–101.45 mg/100 g FW) and of a well-balanced ω-6 to ω-3 fatty acid ratio (3:1) with USO 31, which showed the best phytochemical profile and consequently the best antioxidant activity (about two times higher than Futura 75). The HSMs still retained part of the phytochemicals identified in the HSOs (polyphenols, tocopherols, and the preserved ω-6/ω-3 fatty acids ratio) and a modest antioxidant activity. Furthermore, they showed a very interesting nutritional profile, which was very rich in proteins (29.88–31.44 g/100 g FW), crude fibers (18.39–19.67 g/100 g), and essential and non-essential amino acids. Finally, only a restrained amount of anti-nutritional factors (trypsin inhibitors, phytic acid, and condensed tannins) was found, suggesting a promising re-use of these byproducts in the nutraceutical field.

## 1. Introduction

*Cannabis sativa* L., commonly known as hemp, is an herbaceous plant belonging to the Cannabaceae family. It has a long history of cultivation, which makes it difficult to identify its exact center of origin. According to molecular and DNA sequencing studies, most researchers agree that this plant is native to Western and Central Asia and that it has also been cultivated commercially in Europe and in parts of China, Japan, Canada, and USA [1,2]. Indeed, from this highly versatile plant, it is possible to obtain various products of industrial interest such as fiber, bio-building and thermal insulated materials, seeds, flour, and vegetable oil (hempseed oil, HSO) with important nutritional and functional features as well as bioactive compounds of pharmacological interest [3,4,5,6,7]. Nowadays, due to the increased knowledge about their high nutritional value and potential use in pharmaceuticals, there is a growing interest in the seeds belonging to several non-psychotropic *C. sativa* varieties containing less than 0.3% Δ^9^-tetrahydrocannabinol (Δ^9^-THC) [8]. 

HSO is one of the few vegetable oils that contains about 80% polyunsaturated fatty acids in a perfect 3:1 ratio of ω-6 to ω-3, which is suggested as optimal for human nutrition for the potential health benefits [9,10,11]. An unfortunate paradox of HSO resides within its unsaturation that makes it both highly nutritious but chemically unstable [12]. Differences in stability between highly unsaturated vegetable oils can be observed, and are at least partially attributable to the proportions of co-expressed ancillary components [13]. These include phenolics and tocopherols that act as strong antioxidants. The etiopathology of numerous chronic diseases involves oxidative damage to cellular components. Considering this, minimizing oxidative damage may be one of the most important approaches to the primary prevention of chronic diseases and ageing-associated health problems. Antioxidants have been detected in several plants and foods, including seed oils [14,15]. HSO shows excellent oxidative stability, suggesting the possible presence of secondary metabolites with strong antioxidant activity [6,16]. The *C. sativa* plants brought to cultivation to obtain seeds as well as their derivatives, commonly called “industrial hemp”, must be listed in the European Union (EU) Common Catalogue of Varieties of Agricultural Plant Species, and must be characterized by a Δ^9^-THC value ≤ 0.3. Cold-press processing is recommended for HSO extraction because it preserves its rich nutritional profile, packing numerous powerful benefits for the human body. This vegetable oil has higher quantities of antioxidants, essential omega fatty acids and vitamins in comparison with the same heat-treated seed oil. Considering that less than 10% of the world’s population gets the recommended daily amount of vitamins and minerals, cold-pressed HSO, for its nutritional benefits, might be a solution. Moreover, after cold pressing, a high-protein hempseed meal (HSM) remains [17] that can be classified as a functional food due to its valuable nutritional properties. The composition of this waste product is strictly dependent on various factors including genetics, environmental, and processing techniques [18]. Edestin and albumin are the two main proteins in hempseed. Furthermore, they are rich in sulfur amino acids and especially in arginine and glutamic acid. Hempseeds are well known for their bioactive properties related to vitamin D, optimizing calcium and bone metabolism through their metabolite 1,25-dihydroxycholecalciferol. Moreover, recent research suggests that they help to improve lower limb muscle strength and lean mass in humans [19].

To date, there are no studies that simultaneously evaluate the nutritional and health profile of HSOs and their waste products. Furthermore, some Cannabis strains are still poorly understood or have never been studied before. The Futura 75 and USO 31 hemp varieties are gaining popularity for many applications. They are able to acclimate to both northern and southern climates, and in particular southern climates can extend growing cycles, resulting in good yields of seeds with a high oil content (28–30%). 

Considering this, the aim of the present study was to evaluate and compare, for the first time, the phytochemical profile and the antioxidant activity of the two cold-pressed seed oils from Futura 75 and USO 31 (Δ^9^-THC ≤ 0.2%), as well as the nutritional, anti-nutritional, phytochemical, and biological properties of their main waste product (HSM) in order to hypothesize their possible re-use, in a circular economy perspective, in the nutraceutical field.

## 2. Results

### 2.1. Quality Parameters of Hempseed Oil

The nutritional features of the HSOs were evaluated by checking the quality parameters reported in Table 1. Both HSOs showed very interesting values with low acidity (≤0.65%) and peroxide index (≤1.88 mg O_2_/Kg) values, as well as dienes and trienes content (K_232_ ≤ 0.75 and K_270_ ≤ 0.18, respectively) which allowed us to calculate a very low ΔK index (≤0.008), which fits perfectly within the range of a high-quality extra virgin olive oil.

### 2.2. Nutritional Profile and Determination of Anti-Antritional Factors of HSMs

The aim of this study was also to demonstrate that the waste products obtained after the cold pressing of hempseed, i.e., hempseed meals, can be recovered and used from a nutritional and healthy point of view in a circular economy perspective. To this end, after determining the phytochemicals present, the characterization of the nutritional profile and the determination of potential anti-nutritional factors such as trypsin inhibitors, phytic acid, and condensed tannins were carried out. 

USO 31 and Futura 75 HSMs are excellent sources of proteins (~31 g/100 g FW) and crude fibers (~19 g/100 g FW), with a modest content of lipids (~9.5 g/100 g FW) and a very low amount of sugars (~1.3 g/100 g FW). Furthermore, they contain modest amounts of phytic acid (7 mg/g FW), trypsin inhibitors (~0.5 TIU/mg protein), and condensed tannins (0.015 mg/g FW), thus showing a very interesting and highly bioaccessible nutritional profile (Table 2).

### 2.3. Phytochemical Analyses

As a first step of phytochemical characterization, two colorimetric tests were performed for the determination of the total phenols and flavonoids. The HSOs showed, in both cases, the highest values in terms of total phenols (384.52 ± 23.55 and 290.32 ± 10.49 mg/g FW, for USO 31 and Futura 75, respectively) and flavonoids (334.70 ± 25.66 and 224.76 ± 11.60 mg/g FW, for USO 31 and Futura 75, respectively) with USO 31, which proved to be the one richest in polyphenols. Although a marked reduction in both values was observed in the HSMs (total phenols 16.32 ± 1.15 and 2.88 ± 0.07 mg/g FW; total flavonoids 2.96 ± 0.09 and 2.30 ± 0.02 mg/100 g FW, for USO 31 and Futura 75, respectively), it is interesting to observe a superimposable behavior with the USO 31 HSM, which still showed the highest polyphenol content after cold pressing. 

**Figure 1:**
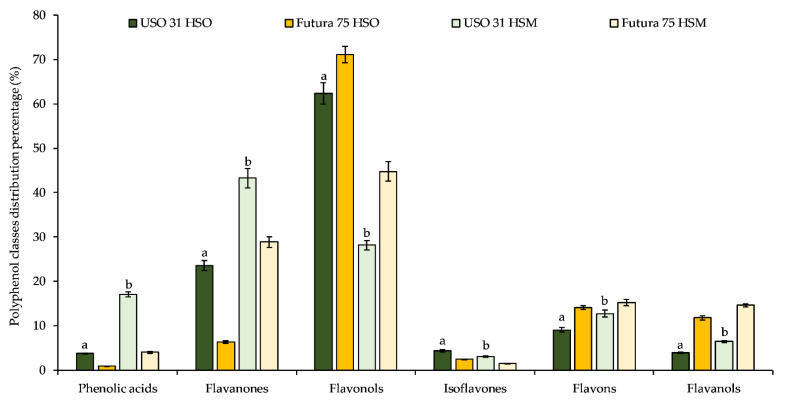
Percentage distribution of the different classes of polyphenols identified in USO 31 and Futura 75 hempseed oil (HSO) and hempseed meal (HSM). ^a^ *p* < 0.05 vs. Futura 75 HSO; ^b^ *p* < 0.05 vs. Futura 75 HSM.

These results were also confirmed by LC-DAD-ESI-MS analysis (Figures S1–S4), which showed a higher total polyphenol content for USO 31 compared to Futura 75, both in regard to the HSOs (125.24 vs. 80.48 µg/100 g FW) and HSMs (44.58 vs. 37.89 µg/100 g FW). Furthermore, as can be seen from Table 3 and Figure 1, a different phytochemical profile was found in terms of the abundance of the different polyphenol classes identified.

Specifically, the USO 31 HSO showed a clear prevalence of flavonols (62.40%), followed by flavanones (23.57%), flavones (9.07%), isoflavones (4.39%), flavanols (3.94%), and phenolic acids (3.78%). On the contrary, the Futura 75 HSO, although it also showed a high content of flavonols (71.12%), even higher than USO 31, showed a different profile, in percentage terms, of the other classes of polyphenols, with the flavones as the most representative (14.12%) followed by flavanols (11.77%), flavanones (6.32%), isoflavones (2.53%), and phenolic acids (0.91%). The percentage distribution of the various classes of polyphenols changes markedly by evaluating the waste products after cold pressing. The USO 31 HSM appears to be characterized by a very abundant presence of flavanones (43.27%), followed by flavonols (28.20%), phenolic acids (17.11%), flavones (12.74%), flavanols (6.40%), and isoflavones (3.08%). Conversely, the Futura 75 HSM was very rich in flavonols (44.77%), followed by flavanones (28.90%), flavones (15.19%), flavanols (14.63%), phenolic acids (4.01%), and isoflavones (1.49%). In both varieties, the most abundant flavonoid was the quercetin-3-*O*-rutinoside or rutin (77.89 and 56.93 µg/100 g FW for USO 31 and Futura 75, respectively), followed by eriodictyol-7-*O*-glucoside and eriodictyol (14.87 and 14.33 µg/100 g FW) in the USO 31 HSO, and by catechin and apigenin (9.43 and 6.04 µg/100 g FW) in the Futura 75 HSO. Interesting is the profile of the HSMs, which showed a higher content of naringenin-7-*O*-glucoside (6.95 and 5.85 µg/100 g FW for USO 31 and Futura 75, respectively) and naringenin (3.45 and 3.28 µg/100 g FW for USO 31 and Futura 75, respectively) in comparison with the respective HSOs, probably due to an intense enzymatic activity that occurred during the manufacturing process.

Contrary to what was observed for polyphenols, the Futura 75 HSO showed the best tocopherol content (101.45 vs. 81.69 mg/100 g FW in USO 31), whereas comparable amounts were detected in the HMSs (0.85 vs. 0.86 mg/100 g FW in USO 31) (Table 4). 

As in the most seed oils, the γ-isomer was the most abundant (77.43 and 92.45 mg/100 g FW for USO 31 and Futura 75, respectively), followed by α-, δ-, and β-tocopherol. However, it is interesting to note that the only tocopherol that was present in comparable amounts in the two HSOs was the α-tocopherol (3.92 and 4.77 mg/100 g FW, for USO 31 and Futura 75, respectively), to which the biological activity is mainly ascribable, especially the antioxidant one.

Regarding the fatty acids profile (Table 5), the USO 31 and Futura 75 HSOs showed superimposable behaviors with polyunsaturated fatty acids (PUFAs), which represented about 74% of the total fatty acids, followed by monounsaturated fatty acids (MUFAs, ~15%) and saturated fatty acids (SFAs, ~11%). 

In the USO 31 HSO and HSM, linoleic acid was the most abundant compound (~52%) followed by oleic, linolenic, palmitic, and stearic acid. On the contrary, in the Futura 75 HSO and HSM, linoleic acid (~54%), which always remained the most representative compound, was followed by linolenic, oleic, palmitic, and stearic acid. Among the PUFAs, about 56% were n-6 fatty acids, giving the HSOs and HSMs a well-balanced ω-6 to ω-3 fatty acid ratio (3:1).

Finally, to better investigate the HSMs, a characterization of the amino acid profile was also carried out. Thirteen and fourteen amino acids were detected in the USO 31 and Futura 75 HSMs, of which seven were essential (Table 6). From this point of view, the two varieties showed important differences. USO 31, which showed a higher amino acid content than Futura 75 (22.12 vs. 18.31 g/100 g FW), contained very high amounts of threonine (an essential amino acid, 9.26 g/100 g FW), followed by serine, glycine, tyrosine (essential amino acids), and alanine. On the contrary, Futura 75 contained mainly serine (10.48 g/100 g FW), followed by glycine, threonine (essential amino acids), and glutamic acid. Therefore, it should be noted that USO 31 showed the best amino acid profile, showing an essential amino acid content of 11.52 g/100 g FW, which was ~11 times higher than Futura 75.

### 2.4. Antioxidant and Free-Radical Scavenging Activity

The antioxidant and free-radical scavenging activity is mainly attributable to the concentration of polyphenols and tocopherols present in the extracts under examination. Among the tocopherols, α-tocopherol played a pivotal role. Since in terms of alpha-tocopherol both the USO 31 and Futura 75 HSOs and HSMs showed quite comparable values, what most influences the biological behavior of the extracts is the polyphenol content, which was much higher in the USO 31 variety. Indeed, as reported in Table 7, USO 31 showed the strongest antioxidant and free-radical scavenging activity in all the tests carried out, both in terms of the HSOs and HSMs. The extracts, in particular, showed a marked antioxidant activity in the DPPH test, based on electron and hydrogen atom transfer reactions followed by ORAC, based on a hydrogen atom transfer reaction, FRAP, based on an electron transfer reaction, and TEAC also based on electron and hydrogen atom transfer reactions. Finally, the extracts also showed a fair iron-chelating capacity, mainly attributable to the flavonoidic component. The difference in terms of antioxidant activity between the two investigated varieties is important and statistically significant, being 1.2–1.87 times and 1.36–4 times greater for the USO 31 HSO and HSM, respectively.

Finally, it is interesting to note how the two byproducts continued to show biological activity, mainly attributable to the polyphenolic component which, as reported in Section 2.3 and in particular in Table 3, was about 2–3 times lower compared to the HSOs, while the tocopherols, being lipophilic and therefore preferentially distributing in the HSOs, were almost absent in the HSMs.

## 3. Discussion

The cold-pressed technique is the most commonly used technique to extract commercial HSO, leaving almost half of the total biomass (HSM) as a byproduct. This waste, which represents a huge cost for companies, is a rich source of compounds with a high nutritional value as well as of secondary metabolites that remain in the squeezing residue. In particular, the unsaponifiable fraction of HSO and HSM, which until now received very little attention by researchers, represents the true inherent potential of industrial hemp products for their possible use in the nutraceutical field [20,21]. 

Although HSOs do not have to meet specific quality parameters to be commercialized, often these parameters are compared with those of other vegetable oils, in particular extra virgin olive oil, which is also obtained exclusively by cold pressing. However, Pharmacopoeia establishes a maximum value of 6% and 10 mEq O_2_/kg for HSO acidity and peroxide value, respectively [22]. Acidity in edible oils is indicative of its conservation status and is strictly dependent on several variables including harvesting type, drying process, and storage [23]. Hand-harvested seeds had lower acidity than machine-harvested ones (~ 2% vs. 3%), as well as clean ventilated seeds with respect to the not-cleaned ventilated seeds (~ 3% vs. 6%). Furthermore, it has been reported that drying and storage increases this index by 0.1% and 0.05% per month. In a recent study, the authors observed that Federcanapa samples’ acidity values (1.9–16%) were higher with respect to certified Futura 75 (1.1%) and even more than the Monoica variety (0.81%) [23], which is the one closest to the USO 31 and Futura 75 HSOs (≤0.65%) analyzed in this study. Similar results were also obtained by Izzo and co-workers [24], who analyzed thirteen commercial HSOs belonging to different varieties cultivated in Italy and produced as a monovarietal or blended oil, finding acidity and peroxide index values ranging from 1.3–9.9% and 1.75–7.62 meq O_2_/kg, respectively. Another study reported acidity and peroxide values for HSOs of 1.76% and 1.94 meq O_2_/kg, respectively [25]. Results comparable to those obtained in the present study were instead observed by Al Jourdi et al. [26], who analyzed three HSOs harvested in ecological crops from Romania, finding acidity and peroxide values ranging from 0.5 to 0.75% and 0.6–1.2 meq O_2_/kg, respectively. Furthermore, since the oxidation of PUFAs may generate conjugated dienes (K_232_) and trienes (K_270_), spectrophotometric analyses were carried out to calculate the ΔK value, which was ≤0.008, significantly lower with respect to that previously reported for HSOs (≤0.047) [24]. 

Other than quality parameters, it has been demonstrated that cultivar, variety, geographical origin, harvesting, drying, and storage conditions also affect the expression of minor constituents in HSOs such as total phenols, and the values have been found ranging from 32.50 to 160.80 mg GAE/g [24], which are well below those found in this study (≥ 290 mg GAE/g). On the contrary, a superimposable value was observed for Finola HSO total phenols and flavonoids content [6]. Regarding the total phenol content in HSMs, it has been recently reported that it may range from 0.39 to 0.91 mg/g [27,28]. However, Chen et al. [29] showed higher results in defatted HSM (3.9–15.6 mg GAE/g), with values superimposable to those obtained in the present study (2.88–16.32 mg GAE/g). According to the literature, specimens of the same plant species growing under different environmental conditions show significant differences in the production and accumulation of specific secondary metabolites such as polyphenols [30]. Furthermore, these differences increase based on the growing year and plant genotype. At this purpose, Irakli et al. [4] analyzed seven industrial hemp varieties harvested in three successive growing years, finding lignans and phenolic acids such as protocatechuic, *p*-hydroxybenzoic, and cinnamic acid as the main polyphenols. On the contrary, Faugno et al. [31], investigating if the polyphenol profile of USO 31 hempseed is affected by different cropping techniques, observed that flavonol glycosides were the main and characterizing USO 31 HSO polyphenols, according to our results. 

The same trend was also observed in our previous study on the Finola variety, in which flavonoids were the most abundant polyphenols class HSO [6].

However, the geographical provenience of hemp should be strictly considered in order to select the most suitable variety for a specific nutraceutical purpose, without forgetting that, in any case, the final expression in secondary metabolites is strongly influenced by a whole series of parameters involving growing, harvesting, storage, and oil pressing conditions [31,32].

Together with polyphenols, other secondary metabolites that play a pivotal role in counteracting oxidation events in vegetable oils by stopping radical chain reactions against PUFAs and positively affecting their storage are tocopherols [33,34]. This is also the reason why extra virgin oils have a higher oxidative stability than refined oils. Among tocopherols, the γ-isomer is found to be the most abundant, followed by α-, δ-, and β-isomers, according to previous results [6,35,36]. This is of great importance because, although α-tocopherol is the best antioxidant among the tocopherol isomers, γ-tocopherol was reported to decrease low-density lipoprotein oxidation and to increase superoxide dismutase and nitric oxide synthase activity with a higher efficiency than α-tocopherol [37]. The USO 31 and Futura 75 HSOs investigated in the present study showed a higher total tocopherols content with respect to that previously reported, ranging from 3.47 to 13.25 mg/100 g [6,33]. The closest tocopherols content (63.03–85 mg/100 g) was observed instead by Anwar et al. [38], who analyzed HSOs from three agro-ecological zones of Pakistan. Finally, similar results were obtained by The et al. [25], who found a total tocopherols value of 59.16 mg/100 g. The greatest difference in terms of tocopherols was found in the content of γ-tocopherol, which, in the varieties investigated in this study, was approximately three times greater than that reported in the literature (77.43–92.45 vs. 28.23 mg/100 g) [33]. On the contrary, the α-tocopherol content was very close to the ranges previously reported (1.83–3.53 mg/100 g) [33]. However, it should not be forgotten that even substantial differences in terms of tocopherols can be recorded for the same reasons mentioned above for polyphenols, such as storage time, oxygen exposure, and temperature [39].

The endocannabinoid system (ECS) plays a critical homeostatic role in modulating PUFA signaling to maintain a balanced inflammatory and redox status. Dietary interventions based on animal or plant origin foods rich in PUFAs are increasingly used to support the ECS tone, promoting a healthy metabolism, brain, and emotional well-being and improving the risk factors associated with cardiovascular diseases. For this specific nutraceutical application, HSOs and HSMs represent matrices of primary choice because of their unique ω6:ω3 ratio (3:1), which is recommended for human nutrition and considered to be very important to reduce the risk of arteriosclerosis and coronary heart disease [40,41]. Moreover, the European Commission has authorized the use of hempseed and co-products in animal nutrition as well. Indeed, as demonstrated by a recent in vivo study, hempseed cake is useful in dogs’ nutrition as it leads, after 30 days, to a reduction in liver and renal markers and cholesterol, due to the healthier fatty acid profile [42].

PUFAs indeed are able to exert several health properties such as hypocholesterolemic, anti-hypertensive, anti-inflammatory, immunostimulant, anti-diabetic, cardioprotective, dermo-protective, and anti-obesity effects [36,43,44]. 

According to our results, the main fatty acids in the HSM were linoleic (54.09−55.42%), α-linolenic (17.31−18.42%), and oleic (12.96−13.93%) acid, followed by palmitic (6.48−7.90%), stearic (3.18−3.86%), and γ-linolenic (2.61−2.76%) acid [18]. Similar results were obtained for several commercial HSOs from different industrial hemp varieties, with the main fatty acids (α-linoleic, α- and γ-linolenic, and oleic acid) alone representing 80–90% of the total fatty acids [24,25,33,42,45,46]. Longer chain fatty acids, i.e., C:20 or higher, were also detected, but their concentration was significantly lower according to our results [46]. Furthermore, according to the British Department of Health and WHO/FAO experts, the PUFA/SFA ratio detected in our study (4.34–6.11) falls perfectly, also according to previous results (6.02–7.14) [18], within the recommendations, which suggests values above 0.40 [47,48]. Even in this case, several factors may affect fatty acid content and composition such as cultivars, pedo-climatic conditions, and farming, in particular during seed development [24]. 

All these compounds contribute to determining the antioxidant properties of HSOs and HSMs. Antioxidants play a pivotal role in protecting cell constituents against oxidative damage, which is well known to be the first step for the onset of various chronic diseases [49]. For this reason, antioxidant capacity is widely used as a screening parameter to characterize foods or medicinal plants as well as their bioactive phytochemicals. Currently, few works that evaluate antioxidant and free-radical scavenging activity through an in vitro test set, which are useful to characterize the behavior of extracts in different environments and reaction mechanisms, are available. Comparing the results of the present study with our previous work about the Finola HSO variety, what is immediately evident is that, in accordance with the richer phytochemical profile of the USO 31 and Futura 75 HSOs, the antioxidant activity appears significantly higher (1.1–2.9 times) than that observed for the Finola variety in all the tests carried out. The best antioxidant activity was recorded by the DPPH and ORAC test according to previous results [6,23] because they are generally considered more sensitive than other common antioxidant methods, such as TEAC, although highly variable values ranging from 0.2 to 22.3 mM TE/g were recorded [50]. Since the antioxidant activity in addition to fatty acids is mainly attributable to the polyphenols and tocopherols content, it is evident how the parameters that influence the expression and accumulation of these bioactive compounds, such as harvesting and storage, also consequently influence antioxidant activity. Recently, the antioxidant potential of HSOs was also demonstrated in vivo by Vitorović et al. [51], who analyzed the effects of HSOs on oxidative stress markers and on the life cycle of *D. melanogaster* under non-stress and hydrogen peroxide-induced stress conditions, demonstrating how the antioxidant effect is closely related to the HSO dose. On the contrary, regarding the antioxidant properties of HSMs, it has been recently demonstrated that, in the circular economy perspective of the re-use of hempseed byproducts for nutraceutical purposes, the antioxidant activity of HSM, already reduced with respect to the HSO, generally decreases after extrusion [28]. This result might have the same explanation as the reduction in the total phenols and flavonoids content, mainly due to the high screw speed and barrel moisture. Indeed, the friction generated during extrusion increases the internal product’s temperature, therefore leading to phenolics loss. Furthermore, it seems that this effect is amplified with ingredients rich in proteins and carbohydrates [28].

Regarding the nutritional profile of HSMs, according to our results, the moisture content ranged from 6.98% to 7.88% [18]. Proteins were the most abundant nutrient (10.62–44.36%), followed by lipids (8.26–18.60%) and sugars (0–4.96%). However, the greatest variability was recorded in the crude fiber content with values that could reach 29.54% [18]. 

Interestingly, the anti-nutrients factors in the USO 31 and Futura 75 HSMs analyzed in this study were well below those determined in a previous work on HSMs of the Helena variety [18] and those obtained for watermelon, pumpkin, and paprika seed flour [52]. Finally, the phytic acid content was lower than that of Canola meal, as reported by Bell et al. [53], and of the HSM of Italian and French varieties [54]. This is very important because the presence of certain anti-nutrients may limit the recovery of HSM as edible-grade products in human nutrition, as they influence protein digestibility, organoleptic properties, and the bioavailability of macro- and microelements. However, when evaluating the consumption of protein-rich meals, the bioavailability of amino acids must always be evaluated. Indeed, a recent study, which investigated and compared the postprandial events related to satiety and protein metabolism following the acute consumption of high-protein meals of animal and plant origins, demonstrated important differences in the bioavailability of amino acids, suggesting an important and favorable impact of the food matrix in plant-based meals compared to meat-based ones [55]. Amino acids are emerging as new biomarkers for metabolic disorders; just think that phenylalanine and tyrosine have been associated with insulin resistance in men [56] and that alanine, glutamine, glycine, as well as other amino acids such as histidine, arginine, and tryptophan did not show any association with insulin resistance. High dietary intakes of branch-chain amino acids (BCAAs) have been associated with type two diabetes (T2D), suggesting that nutritional strategies that help to maintain a lower concentration of these amino acids could be used in T2D prevention. Even in this case, however, it has been shown that BCAA intake from plant sources has a lower impact on health. From this point of view, the HSMs analyzed in the present study had a very interesting amino acid profile, being rich, in particular the USO 31 variety, in essential amino acids and poor, in general, in BCAAs. Furthermore, from the tested meals in the above study, hemp had the highest fiber and total fat content, which exert a pivotal role in the up-regulation of glucagon-like peptide one (GLP-1) gene expression [57] and satiety [55]. Therefore, in the light of the results obtained, USO 31 and Futura 75 HSMs prove to be very promising matrices from a nutritional point of view.

## 4. Materials and Methods

### 4.1. Chemicals

Analytical grade chemicals and reagents, LC and GC grade solvents, a 17 amino acid mix solution, a 37 component fatty acid methyl ester (FAME) mix (both certified reference material, TraceCERT^®^), as well as tocopherol standards (α-δ) were purchased from Merck (Darmstadt, Germany). The reference compounds of the polyphenols reported in Table 3 were purchased from Extrasynthese (Genay, France). 

### 4.2. Sample Recovery and Processing

Cold-pressed HSOs from the Futura 75 and USO 31 varieties of industrial hemp (*Cannabis sativa* L., Δ^9^-THC ≤ 0.2%) as well as their HSMs were kindly provided by Scotto & D’Aulerio (Sativa Molise, Italy). The pressing of the seeds was made with a screw press at room temperature. For both varieties, the data of the input (hempseed) and output (oil and pressing meal) as well as the pressing time were equivalent, by obtaining a sample recovery of 20–30% and 70–80% for HSO and HSM, respectively, for both varieties. 

For preliminary phytochemical screening (total phenols and flavonoids) and LC-DAD-ESI-MS analysis of the polyphenols, the HSOs and HSMs were processed according to Smeriglio et al. [6] obtaining polyphenol-rich extracts (HSOE and HSME, respectively) that were also used for antioxidant activity evaluation. Briefly, 10 g of HSOs and HSMs were added to 20 mL of a methanol/water mixture (8:2 *v*/*v*) five times. The supernatants were collected and concentrated in the dark under vacuum by a rotary evaporator (Buchi R-205, Cornaredo, Italy) at room temperature (RT) until syrup consistency. Extracts were added to 10 mL of acetonitrile and defatted thrice with 10 mL of hexane. After this, the samples were brought to dryness with a gentle stream of nitrogen and were stored at −20 °C until the subsequent analyses (mean extraction yield 5.60% and 2.40% for the HSO and HSM, respectively). The determination of the nutritional and anti-nutritional factors, as well as of fatty acids, amino acids, and tocopherols was carried out directly on the starting materials (HSOs and HSMs), treating the samples as specified in the following sections. 

### 4.3. Quality Parameters of Hempseed Oil

The quality parameters of the HSO (acidity, peroxide value, conjugated dienes and trienes, and ΔK) were evaluated according to the analytical procedures reported in the European Community Regulation (EEC) 2568/91 and subsequent amendments [58].

### 4.4. Phytochemical Screening

#### 4.4.1. Total Phenols

The total phenols were quantified according to Smeriglio et al. [6]. Briefly, 50 µL of the USO 31 and Futura 75 HSOEs and HSMEs (1.25–10 mg/mL and 10–80 mg/mL, respectively) were added to 450 µL of deionized water and 500 µL of Folin–Ciocalteu reagent. After 3 min, 500 µL of 10% sodium carbonate was added and the samples were left in the dark at RT for 1 h, vortex mixing every 10 min. Absorbance was read at 785 nm with a UV–Vis spectrophotometer (Model UV-1601, Shimadzu, Kyoto, Japan) against a blank consisting of the same sample solvent (methanol). Gallic acid (75.0–600 µg/mL) was used as the reference compound and the results were expressed as mg gallic acid equivalents (GAE)/100 g fresh weight (FW). 

#### 4.4.2. Total Flavonoids

The total flavonoids were quantified according to Smeriglio et al. [6]. Briefly, 0.2 mL of the USO 31 and Futura 75 HSOEs and HSMEs (1.25–10 mg/mL and 10–80 mg/mL, respectively) were mixed with 0.2 mL of AlCl_3_ and 1.2 mL of sodium acetate (2 mg/mL and 50 mg/mL, respectively) and were incubated for 2.5 h at RT. The absorbance was recorded at 440 nm by using the same instrument and blank reported in Section 4.4.1. The results were expressed as mg quercetin equivalents (QE)/100 g FW. 

### 4.5. Polyphenols Determination by LC-DAD-ESI-MS Analysis

A polyphenol characterization of the USO 31 and Futura 75 HSOEs and HSMEs was carried out according to Smeriglio et al. [59] by LC-DAD-ESI-MS analysis. At this purpose, a Luna Omega PS C18 column (150 mm x 2.1 mm, 5 µm; Phenomenex, Torrance, CA, United States) and a mobile phase consisting of 0.1% formic acid (Solvent A) and methanol (Solvent B) were used according to the following elution program: 0–3 min, 0% B; 3–9 min, 3% B; 9–24 min, 12% B; 24–30 min, 20% B; 30–33 min, 20% B; 33–43 min, 30% B; 43–63 min, 50% B; 63–66 min, 50% B; 66–76 min, 60% B; 76–81 min, 60% B; 81–86 min, 0% B and equilibrated for 4 min. The injection volume was 5 µL and the column oven was set at 25 °C. UV–Vis spectra were recorded ranging from 190 to 600 nm and chromatograms were acquired at different wavelengths (220, 260, 292, 330, and 370 nm) to identify phenolic acids and all flavonoid classes. Mass spectrometer (ion trap, model 6320, Agilent Technologies, Santa Clara, CA, USA) parameters, operating in a negative (ESI-) and positive (ESI+) ionization mode, were set as follows: 3.5 kV capillary voltage, 40 psi nebulizer (N_2_) pressure, 350 °C drying gas temperature, 9 L/min drying gas flow, and 40 V skimmer voltage. Acquisition was carried out in full-scan mode (90–1000 m/z). Data were acquired by Agilent ChemStation software version B.01.03 and Agilent trap control software version 6.2. 

The identification of all the polyphenols reported in Table 3 was carried out by comparing their UV–Vis spectra, retention time, and mass spectrum with commercially available standards. Quantification was performed by building external standard calibration curves. The results were expressed as µg of each polyphenol/100 g of FW. A limit of detection (LOD) ≤ 1 ng/mL was calculated.

### 4.6. Determination of Tocopherols by HPLC-FLD Analysis

The tocopherol profile was evaluated according to the UNI EN ISO 9936:2011 Official Method [60]. Briefly, 100 mg of the USO 31 and Futura 75 HSOs were solubilized in 10 mL of *n*-heptane, whereas the HMSs were added to 10 mL of *n*-heptane, vortex-mixed, and centrifuged at 3000× *g*, 4 °C three times. The supernatant was collected, concentrated to dryness in the dark under vacuum by a rotary evaporator (Buchi R-205, Cornaredo, Italy) at RT, and then resuspended in 10 mL of *n*-heptane. After this, both the HSO and HSM heptane solutions were filtered through a 0.22 µm nylon syringe filter and injected (10 µL) into the Agilent HPLC system (1100 series, Santa Clara, CA, USA), equipped with a fluorescence detector (FLD) (G1321). Chromatographic elution was performed with a LiChrosorb SI-60 column (250 mm x 4.6 mm, 5 µm; Phenomenex, Torrance, CA, United States) maintained at 25 °C by using an *n*-heptane/tetrahydrofurane (96.15/3.85 *v*/*v*) mixture as the mobile phase with a flow rate of 1.0 mL/min. Fluorescence detection (λ_ex_ 295 nm, λ_em_ 330 nm) was used to identify and quantify the α-, β-, γ-, and δ-tocopherol content by using external standard calibration curves. The results were expressed as mg of each tocopherol/100 g FW. A LOD ≤ 10 pg/mL was calculated.

### 4.7. Determination of Fatty Acids by GC-FID and GC–MS Analysis

The fatty acid profile was evaluated according to Smeriglio et al. [61]. Briefly, the USO 31 and Futura 75 HSOs and HSMs were treated with a chloroform/methanol (2:1, *v*/*v*) mixture and were dried by a gentle stream of nitrogen at RT. Transesterification with a 14% boron (III) fluoride methanol solution was carried out to obtain FAMEs. Gas chromatographic (GC) analysis was performed on an Agilent gas chromatograph (7890A) equipped with a flame ionization detector (FID) (Agilent Technologies Santa Clara, CA, USA). Elution was carried out with an HP-5MS capillary column (30 mm, 0.25 mm coated with 5% diphenyl- and 95% dimethyl-polysiloxane, 0.25 µm film thickness) using helium as the carrier gas (1 mL/min, constant flow). The injection was conducted in split mode (50:1), with an injected volume of 1 µL. The injector and detector temperatures were set at 250 °C and 280 °C, respectively. The oven temperature was held at 50 °C for 2 min, increased to 250 °C (4 °C/min), and maintained at 250 °C for 15 min. The percentages of compounds were determined from their peak areas in the GC-FID profiles. Gas chromatography–mass spectrometry (GC–MS) analysis was carried out on the above instrument, coupled with an Agilent 5975C mass detector, with the same column and the same operative conditions used for the GC-FID analysis. The ionization voltage was set to 70 eV, the electron multiplier to 900 V, and the ion source temperature to 230 °C. Mass spectra were acquired in scan mode (*m*/*z* 45–450). 

Detected compounds were identified based on the following parameters: the GC retention index (relative to C7–C40 *n*-alkanes on the HP-5MS column), matching of mass spectra with those reported in the MS library (NIST 08), comparison of MS fragmentation patterns with those reported in the literature, and co-injection with a Supelco 37 component FAME mix (see Section 4.1). A LOD ≤ 10 ng/mL was calculated.

### 4.8. Determination of Amino Acid Profile by HPLC-FLD Analysis

A total amino acid determination was carried out on the USO 31 and Futura 75 HSM samples prepared according to the open hydrolysis method reported in the European commission directive 98/64/EC [62]. The obtained sample solutions were filtered by 0.45 µm of nylon syringe filter, derivatized (1:1, *v*/*v*) with o-phthalaldehyde (OPA) reagent according to Frank et al. [63] and analyzed (10 µL) by the same HPLC-FLD instrument reported in Section 4.6. Separation was performed with a Kinetek XB-C18 column (150 mm x 4.6 mm, 5μm; Phenomenex, Torrance, CA, United States) maintained at 22 °C by using a mobile phase consisting of a 30 mmol/L potassium dihydrogen phosphate buffer with 0.4% tetrahydrofuran adjusted to pH 7.0 with 4 mol/L KOH (Solvent A), and an acetonitrile/water mixture 50:50, *v/v* (Solvent B) with a flow rate of 0.5 mL/min and according to the following gradient elution program: 0 min, 100% A; 0–22 min 52% A; 22–34 min 40% A; 34–35 min 100% A; 35–40 min 100% A.

Fluorescence detection (λ_ex_ 340 nm, λ_em_ 455 nm) was used to identify and quantify essential and non-essential amino acids by using external standard calibration curves. The results were expressed as g of each amino acid/100 g FW. A LOD ≤ 100 pg/mL was calculated.

### 4.9. Nutritional Profile and Anti-Nutritional Factors of Hempseed Meal

The moisture content of the HSMs was determined by oven drying to a constant mass at 105 °C. The protein, lipid, total sugars, ash, and crude fiber content was determined according to AOAC standard methods [64].

Phytic acid was extracted from the HSMs with 0.2 M of HCl and was determined according to Haug and Lantzsch [65]. Tannins were extracted from the HSMs with 70% acetone, evaporated to dryness with a gentle stream of nitrogen, and then resuspended in methanol. Condensed tannins were determined by the vanillin method by using catechin as the reference standard [66]. Trypsin inhibitors were extracted from the HMSs with 0.01 M of NaOH (pH 9) and their activity was measured according to Hamerstrand et al. [67] by using N-benzoyl-DL-arginine *p*-nitroanilide hydrochloride (BAPA) as the trypsin substrate. One trypsin inhibitor unit (TIU) was defined as a 0.01 decrease in absorbance at 410 nm under the assay conditions compared with the negative control (without an inhibitor).

### 4.10. Antioxidant and Free-Radical Scavenging Activity

#### 4.10.1. DPPH Assay

The radical scavenging activity against DPPH was evaluated according to Smeriglio et al. [6]. Briefly, 37.5 µL of HSOEs and HSMEs (0.63–5.0 mg/mL and 1.25–10 mg/mL, respectively) were added to 1 mM of a fresh DPPH methanol solution, vortex-mixed for 10 s, and incubated in the dark at RT for 20 min. Absorbance was recorded at 517 nm by using the same instrument and was blank reported in Section 4.4.1. Trolox was used as reference compound (0.1–0.8 µM).

#### 4.10.2. TEAC Assay

The radical scavenging activity against ABTS was evaluated according to Smeriglio et al. [6]. The reaction mixture, consisting of 4.3 mM of K_2_S_2_O_8_ and 1.7 mM of the ABTS solution (1:5 *v/v*), was incubated for 12 h in the dark at RT, diluted just before the analyses until an absorbance of 0.7 ± 0.02 (734 nm), and was used within 4 h. Fifty microliters of HSOEs and HSMEs (3.13–25 mg/mL and 62.5–500 mg/mL, respectively) were added to 1 mL of the reaction mixture and were incubated at RT for 6 min. The absorbance was recorded at 734 nm by using the same instrument and blank reported in Section 4.4.1. Trolox was used as reference compound (50–400 µM).

#### 4.10.3. FRAP Assay

The ferric reducing antioxidant power was evaluated according to Smeriglio et al. [6]. Fifty microliters of HSOEs and HSMEs (0.63–5.0 mg/mL and 25–200 mg/mL, respectively) were added to 1.5 mL of fresh pre-warmed (37 °C) working FRAP reagent (300 mM buffer acetate pH 3.6, 10 mM 2,4,6-Tris (2-pyridyl)-s-triazine (TPTZ)-40 mM HCl, and 20 mM FeCl_3_) and were incubated for 4 min at RT in the dark. The absorbance was recorded at 593 nm by using the same instrument and blank reported in Section 4.4.1. Trolox was used as reference compound (50–400 µM).

#### 4.10.4. ORAC Assay

The radical scavenging activity against AAPH was evaluated according to Smeriglio et al. [6]. Briefly, 20 µL of HSOEs and HSMEs (1.25–10.0 mg/mL and 25.0–200.0 mg/mL, respectively) diluted in 75 mM of phosphate buffer pH 7.4, were added to 120 µL of fresh 117 nM fluorescein and were incubated for 15 min at 37 °C. Sixty microliters of 40 mM of AAPH radicals were added to start the reaction, which was monitored every 30 s for 90 min (λ_ex_ 485; λ_em_ 520) by a fluorescence plate reader (FLUOstar Omega, BMG LABTECH, Ortenberg, Germany) against the same blank reported in Section 4.4.1. and by using trolox as reference compound (10–100 µM).

#### 4.10.5. Iron-Chelating Activity

The iron-chelating activity was evaluated as described by Smeriglio et al. [6]. Fifty microliters of HSOEs and HSMEs (2.5–20.0 mg/mL and 50.0–400.0 mg/mL, respectively) were added to 25 μL of 2 mM FeCl_2_ 4 H_2_O and were incubated at RT for 5 min. After that, 50 μL of 5 mM ferrozine and 1375 μL of deionized water were added to the reaction mixture. The absorbance was recorded after 10 min at 562 nm by using the same instrument and blank reported in Section 4.4.1. Ethylenediaminetetraacetic acid (EDTA) was used as reference compound (10–100 µM).

### 4.11. Statistical Analysis

The results were expressed as the mean ± standard deviations (SD) of three independent experiments in triplicate (*n* = 3) and were analyzed by one-way analysis of variance (ANOVA) followed by Student–Newman–Keuls and Tukey’s test by SigmaPlot 12.0 software (Systat Software Inc., San Jose, CA, USA). *p* ≤ 0.05 was considered statistically significant.

## 5. Conclusions

In conclusion, the present study demonstrates how the phytochemical and nutritional profile of HSOs and HSMs is strictly correlated to the hempseed variety considered, to its geographical origin, as well as to the treatment and storage conditions of the finished products. Both the USO 31 and Futura 75 HSOs showed a very interesting phytochemical profile rich in polyphenols, in particular flavonols, tocopherols, mostly γ-tocopherol, and fatty acids in a perfect ω6:ω3 ratio (3:1). The HSM of the two varieties, which showed, albeit reduced, a superimposable phytochemical profile compared to the HSOs, proved to be particularly interesting from a nutritional point of view, with a very high protein and crude fiber content, a low lipid and sugar content, and a very interesting amino acid profile rich in essential amino acids and poor in branched-chain amino acids, which suggests their potential re-use, from a circular economy perspective, in the nutraceutical field.

## Figures and Tables

**Table 1:** Quality parameters of USO 31 and Futura 75 hempseed oil (HSO).

Parameter	USO 31	Futura 75
Acidity (oleic acid %)	0.58 ± 0.02 ^a^	0.65 ± 0.03
Peroxide index (mEq. O_2_/Kg)	1.55 ± 0.04 ^a^	1.88 ± 0.02
Conjugated dienes (K_232_)	0.54 ± 0.02 ^a^	0.75 ± 0.01
Conjugated trienes (K_270_)	0.12 ± 0.00 ^a^	0.18 ± 0.00
ΔK	0.003 ± 0.00 ^a^	0.008 ± 0.00

^a^*p* < 0.05 vs. Futura 75 HSO.

**Table 2:** Determination of nutrient and anti-nutrient compounds in USO 31 and Futura 75 hempseed meal (HSM). Data are expressed as g/100 g of fresh weight (FW) and represent the mean ± standard deviations (SD) of three independent experiments (*n* = 3).

Nutrients (g/100 g FW)	USO 31 HSM	Futura 75 HSM
Moisture	5.76 ± 0.03 ^a^	6.65 ± 0.04
Protein	31.44 ± 0.25 ^a^	29.88 ± 0.18
Lipid	9.21 ± 0.27 ^a^	9.74 ± 0.08
Total sugar	1.18 ± 0.02 ^a^	1.32 ± 0.03
Ash	5.88 ± 0.03 ^a^	6.47 ± 0.04
Crude fiber	19.67 ± 0.14 ^a^	18.39 ± 0.22
**Anti-nutrients**		
Trypsin inhibitors (TIU ^§^/mg of protein)	0.43 ± 0.01 ^a^	0.65 ± 0.03
Phytic acid (mg/g FW)	5.62 ± 0.02 ^a^	8.74 ± 0.04
Condensed tannins (mg/g FW)	0.01 ± 0.00 ^a^	0.02 ± 0.00

^§^ TIU, trypsin inhibitor unit; ^a^ *p* < 0.05 vs. Futura 75 HSM.

**Table 3:** Qualitative and quantitative characterization of polyphenols in methanol extract of USO 31 and Futura 75 hempseed oil (HSO) and their processing byproducts (hempseed meal, HSM) by LC-DAD-ESI-MS analysis. Data, which are the mean ± standard deviations (SD) of three independent experiments (*n* = 3), were expressed as µg/100 g of fresh weight (FW).

Compounds	*n*	R_t_ (min)	λ_max_ (nm)	MS (*m*/z) [M–H]^−^	USO 31 HSO	USO 31 HSM	Futura 75 HSO	Futura 75 HSM
*Hydroxybenzoic acids*								
Gallic acid	1	6.68	268	169	0.58 ± 0.01 ^a^	1.00 ± 0.03 ^b^	0.03 ± 0.00	<LOD
Protocatechuic acid	2	13.20	258; 293	153	0.04 ± 0.00	1.30 ± 0.02 ^b^	0.04 ± 0.00	<LOD
4-Hydroxybenzoic acid	3	23.25	253	137	2.37 ± 0.04 ^a^	1.66 ± 0.01 ^b^	0.03 ± 0.00	0.03 ± 0.00
Vanillic acid	5	31.19	262; 291	167	1.44 ± 0.01 ^a^	0.69 ± 0.01 ^b^	0.02 ± 0.00	<LOD
*Hydroxycinnamic acids*								
Chlorogenic acid	6	31.756	291; 319	353	0.05 ± 0.00 ^a^	<LOD	0.04 ± 0.00	<LOD
*trans*-*p*-Cumaric acid	8	42.575	309	163	0.12 ± 0.00 ^a^	2.18 ± 0.05 ^b^	0.01 ± 0.00	<LOD
*Flavanones*								
Eriodictyol-7-*O*-glucoside	9	47.785	284; 327	449	14.87 ± 0.35 ^a^	1.91 ± 0.02 ^b^	1.23 ± 0.02	<LOD
Naringenin-7-*O*-glucoside	10	53.843	283; 332	433	0.21 ± 0.01 ^a^	6.95 ± 0.03 ^b^	0.18 ± 0.01	5.85 ± 0.04
Eriodictyol	14	60.774	287	287	14.33 ± 0.32 ^a^	6.98 ± 0.02 ^b^	3.57 ± 0.02	1.68 ± 0.01
Naringenin	16	66.997	289	271	0.05 ± 0.00	3.45 ± 0.04 ^b^	0.05 ± 0.00	3.28 ± 0.04
Naringin	17	69.423	284; 330	579	0.06 ± 0.00 ^a^	<LOD	0.05 ± 0.00	<LOD
*Flavonols*								
Quercetin-3-*O*-rutinoside	11	55.292	256; 357	609	77.89 ± 1.55 ^a^	12.51 ± 0.12 ^b^	56.93 ± 0. 84	16.67 ± 0.88
Quercetin-3-*O*-glucoside	12	55.596	254; 354	462	0.07 ± 0.00 ^a^	<LOD	0.11 ± 0.00	<LOD
Kaempferol-3-*O*-rutinoside	13	60.614	266; 350	593	0.06 ± 0.00 ^a^	<LOD	0.08 ± 0.00	<LOD
*Isoflavones*								
Daidzein	15	63.697	248; 302	253	1.96 ± 0.05 ^a^	0.53 ± 0.02 ^b^	1.38 ± 0.01	0.31 ± 0.01
Genistein	18	69.632	261; 332	253	3.54 ± 0.12 ^a^	0.84 ± 0.01 ^b^	0.65 ± 0.02	0.25 ± 0.01
*Flavones*								
Apigenin	19	75.484	268; 330	269	2.54 ± 0.08 ^a^	0.93 ± 0.03 ^a^	6.04 ± 0.05	2.38 ± 0.04
*Flavanols*								
Catechin	4	25.277	279	289	4.88 ± 0.15 ^a^	2.85 ± 0.02 ^a^	9.43 ± 0.08	5.47 ± 0.05
Epicatechin	7	35.944	279	289	0.19 ± 0.00 ^a^	0.79 ± 0.01 ^a^	0.60 ± 0.02	1.17 ± 0.03
Total					125.24	44.58	80.48	37.89

R_t_, retention time; λ_max_, maximum absorbance peak; ^a^ *p* < 0.05 vs. Futura 75 HSO; ^b^ *p* < 0.05 vs. Futura 75 HSM; LOD ≤ 1 ng/mL.

**Table 4:** Determination of tocopherols (α-δ) in USO 31 and Futura 75 hempseeds oil (HSO) and their processing byproducts (hempseed meal, HSM).

Tocopherols	USO 31 HSO	USO 31 HSM	Futura 75 HSO	Futura 75 HSM
	mg/100 g FW			
α-tocopherol	3.92 ± 0.03 ^a^	0.21 ± 0.01 ^b^	4.77 ± 0.14	0.54 ± 0.02
β-tocopherol	0.01 ± 0.00 ^a^	0.20 ± 0.00 ^b^	0.21 ± 0.01	<LOD
γ-tocopherol	77.43 ± 1.22 ^a^	0.46 ± 0.02 ^b^	92.45 ± 1.44	0.31± 0.01
δ-tocopherol	0.32 ± 0.01 ^a^	<LOD	4.02 ± 0.17	<LOD
Total	81.69	0.86	101.45	0.85

^a^*p* < 0.05 vs. Futura 75 HSO; ^b^ *p* < 0.05 vs. Futura 75 HSM^;^ LOD ≤ 10 pg/mL.

**Table 5:** Determination of fatty acids profile in USO 31 and Futura 75 hempseeds oil (HSO) and their processing byproducts (hempseed meal, HSM) by GC-FID and GC–MS analysis. Data, which represent the mean ± standard deviations (SD) of three independent experiments (*n* = 3), are expressed as percentages (%) of total fatty acid methyl esters (FAMEs).

Fatty Acids	USO 31 HSO	USO 31 HSM	Futura 75 HSO	Futura 75 HSM
C14:0–Myristic acid	0.05 ± 0.00 ^a^	0.03 ± 0.00 ^b^	0.02 ± 0.00	0.04 ± 0.00
C16:0–Palmitic acid	8.67 ± 0.05 ^a^	7.77 ± 0.04 ^b^	6.95 ± 0.03	8.04 ± 0.04
C16:1n7–Palmitoleic acid	0.11 ± 0.00	0.10 ± 0.00 ^b^	0.11 ± 0.00	0.14 ± 0.00
C17:0–Heptadecanoic acid	0.05 ± 0.00 ^a^	0.03 ± 0.00 ^b^	0.03 ± 0.00	0.02 ± 0.00
C18:0–Stearic acid	3.76 ± 0.01 ^a^	3.63 ± 0.02 ^b^	2.68 ± 0.03	3.07 ± 0.02
C18:1n9–Oleic acid	16.73 ± 0.13 ^a^	16.43 ± 0.14 ^b^	12.31 ± 0.15	13.15 ± 0.12
C18:2n6–Linoleic acid	51.39 ± 0.25 ^a^	52.78 ± 0.28	56.16 ± 0.33	52.84 ± 0.27
C18:3n6–Linolenic acid	2.03 ± 0.02 ^a^	1.95 ± 0.02 ^b^	2.33 ± 0.05	2.60 ± 0.08
C18:3n3–Linolenic acid	15.36 ± 0.18 ^a^	15.35 ± 0.11 ^b^	17.74 ± 0.16	18.15 ± 0.13
C18:4n3–Stearidonic acid	0.56 ± 0.01 ^a^	0.56 ± 0.01 ^b^	0.81 ± 0.01	0.93 ± 0.02
C20:0–Arachidic acid	0.73 ± 0.02 ^a^	0.79 ± 0.03 ^b^	0.50 ± 0.02	0.57 ± 0.02
C20:1n9–Eicosenoic acid	0.33 ± 0.01 ^a^	0.32 ± 0.02	0.24 ± 0.01	0.28 ± 0.02
C22:2n6–Docosadienoic acid	0.24 ± 0.01 ^a^	0.27 ± 0.01 ^b^	0.14 ± 0.00	0.16 ± 0.00
SFAs	12.53	11.45	9.68	11.17
MUFAs	17.17	16.85	12.65	13.58
PUFAs	70.31	71.70	77.67	75.25
n-3 PUFAs	15.91	15.91	18.55	19.08
n-6 PUFAs	54.39	55.79	59.12	56.18
n-6/n-3 PUFAs	3.42	3.51	3.19	2.94

SFAs, saturated fatty acids; MUFAs, monounsaturated fatty acids; PUFAs, polyunsaturated fatty acids; ^a^ *p* < 0.05 vs. Futura 75 HSO; ^b^ *p* < 0.05 vs. Futura 75 HSM; LOD ≤ 10 ng/mL.

**Table 6:** Determination of amino acids in USO 31 and Futura 75 hempseed meal (HSM) by HPLC-FLD analysis. Data, which represent the mean ± standard deviations (SD) of three independent experiments (*n* = 3), were expressed as g of each amino acid/100 g fresh weight (FW).

Amino Acid	USO 31 HSM	Futura 75 HSM
Essential		
Cysteine	-	0.03 ± 0.00
Histidine	-	0.16 ± 0.01
Threonine	9.26 ± 0.14 ^a^	1.06 ± 0.02
Tyrosine	1.74 ± 0.02 ^a^	0.02 ± 0.00
Valine	0.03 ± 0.00 ^a^	-
Methionine	0.06 ± 0.00 ^a^	0.45 ± 0.02
Phenylalanine	-	-
Leucine	0.38 ± 0.01 ^a^	-
Isoleucine	0.01 ± 0.00 ^a^	0.04 ± 0.00
Lysine	0.04 ± 0.00 ^a^	0.02 ± 0.00
Non-essential		
Aspartic acid	0.09 ± 0.00	0.10 ± 0.01
Glutamic acid	0.08 ± 0.00 ^a^	0.75 ± 0.02
Serine	6.27 ± 0.24 ^a^	10.48 ± 0.28
Glycine	3.13 ± 0.12 ^a^	4.98 ± 0.14
Arginine	-	0.01 ± 0.00
Alanine	1.02 ± 0.01 ^a^	0.18 ± 0.01
Proline	0.01 ± 0.00 ^a^	0.03 ± 0.00

^a^*p* < 0.05 vs. Futura 75 HSM; LOD ≤ 100 pg/mL.

**Table 7:** Antioxidant and free-radical scavenging properties of USO 31 and Futura 75 hempseeds oil (HSO) and their processing byproducts (hempseed meal, HSM). Data, which are the mean ± standard deviations (SD) of three independent experiments (*n* = 3), were expressed as mmoles of trolox or ethylenediaminetetraacetic acid equivalents (TE and EDTA, respectively)/100 g of fresh weight (FW).

Samples	DPPH	TEAC	FRAP	ORAC	Iron-Chelating Activity
	mmoles TE/100 g FW				mmoles EDTA/100 g FW
USO 31 HSO	421.93 ± 10.53 ^a^	1.65 ± 0.11 ^a^	4.04 ± 0.11 ^a^	32.90 ± 0.76 ^a^	0.30 ± 0.00 ^a^
USO 31 HSM	11.54 ± 0.36 ^b^	0.06 ± 0.00 ^b^	0.07 ± 0.00 ^b^	1.45 ± 0.10 ^b^	0.04 ± 0.00 ^b^
Futura 75 HSO	275.53 ± 10.86	1.00 ± 0.01	3.27 ± 0.10	17.53 ± 0.15	0.22 ± 0.00
Futura 75 HSM	6.55 ± 0.44	0.05 ± 0.00	0.04 ± 0.00	0.36 ± 0.00	0.02 ± 0.00

^a^*p* < 0.05 vs. Futura 75 HSO; ^b^ *p* < 0.05 vs. Futura 75 HSM.

## Data Availability

The data presented in this study are available on request from the corresponding author.

## Supplementary Materials

The following supporting information can be downloaded at: https://www.mdpi.com/article/10.3390/molecules27113431/s1. Figure S1. Total ion current chromatogram of USO 31 hempseed oil (HSO). Peak numbers refer to polyphenols reported in Table 3. Figure S2. Total ion current chromatogram of Futura 75 hempseed oil (HSO). Peak numbers refer to polyphenols reported in Table 3. Figure S3. Total ion current chromatogram of USO 31 hempseed meal (HSM). Peak numbers refer to polyphenols reported in Table 3. Figure S4. Total ion current chromatogram of Futura 75 hempseed meal (HSM). Peak numbers refer to polyphenols reported in Table 3.
