# Supplementary material for: Comparative Evaluation of the Nutrients, Phytochemicals, and Antioxidant Activity of Two Hempseed Oils and Their Byproducts after Cold Pressing
Source: Molecules. 2022 May 26;27(11):3431. doi: 10.3390/molecules27113431 (PMC9181874; doi:10.3390/molecules27113431)
Supplement: Supplementary file 1 [file molecules-27-03431-s001.zip › molecules-1725274-supplementary.pdf]

Supplementary material captions

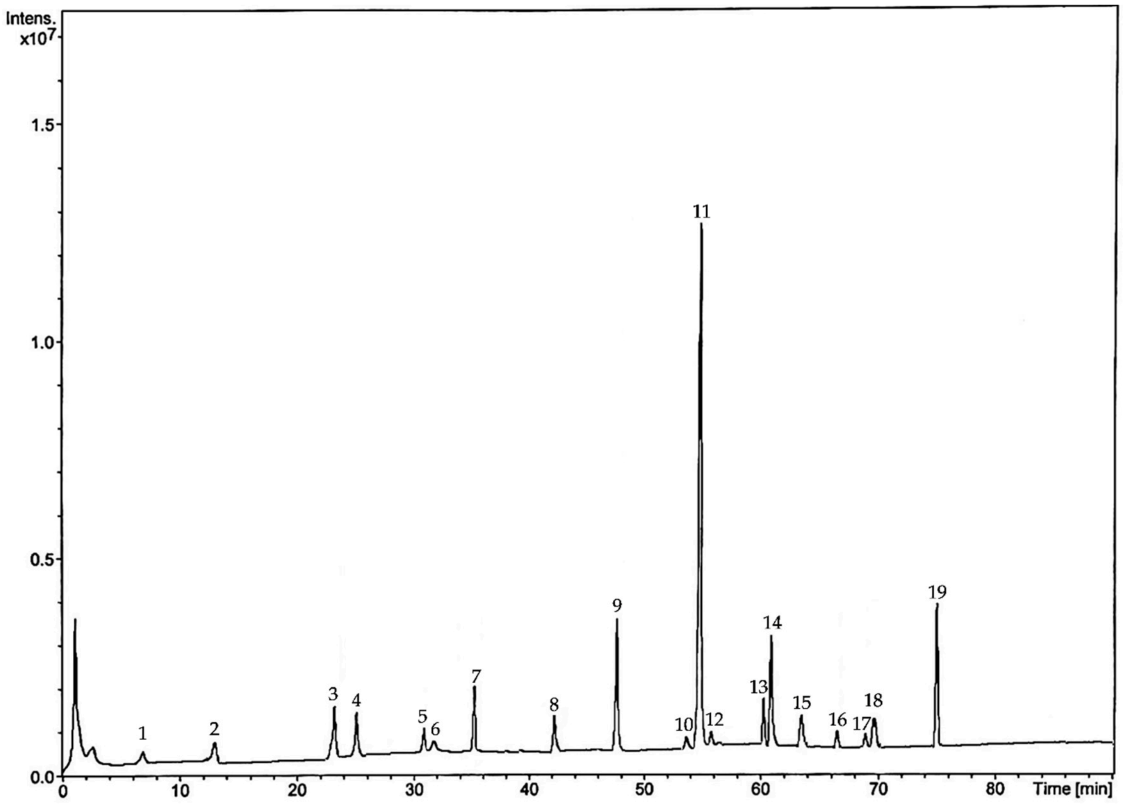

Figure S1. Total ion current chromatogram of USO 31 hempseed oil (HSO). Peak numbers refer to polyphenols reported in Table 3.

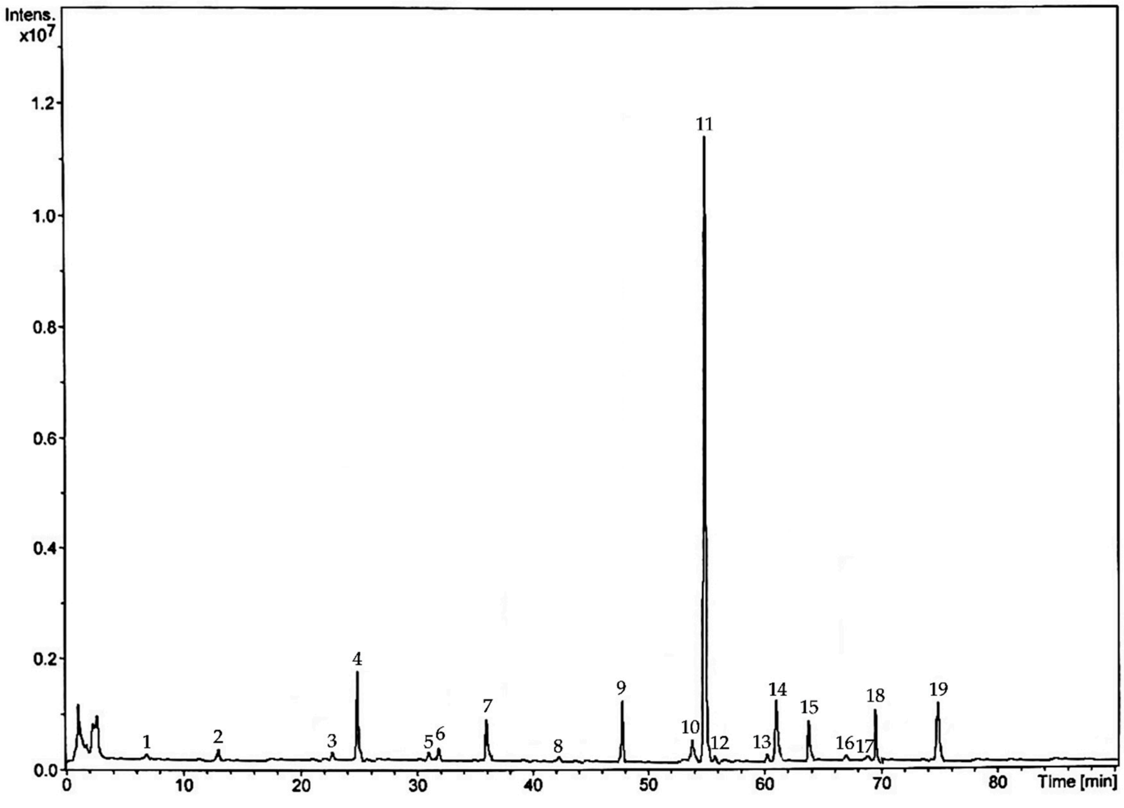

Figure S2. Total ion current chromatogram of Futura 75 hempseed oil (HSO). Peak numbers refer to polyphenols reported in Table 3.

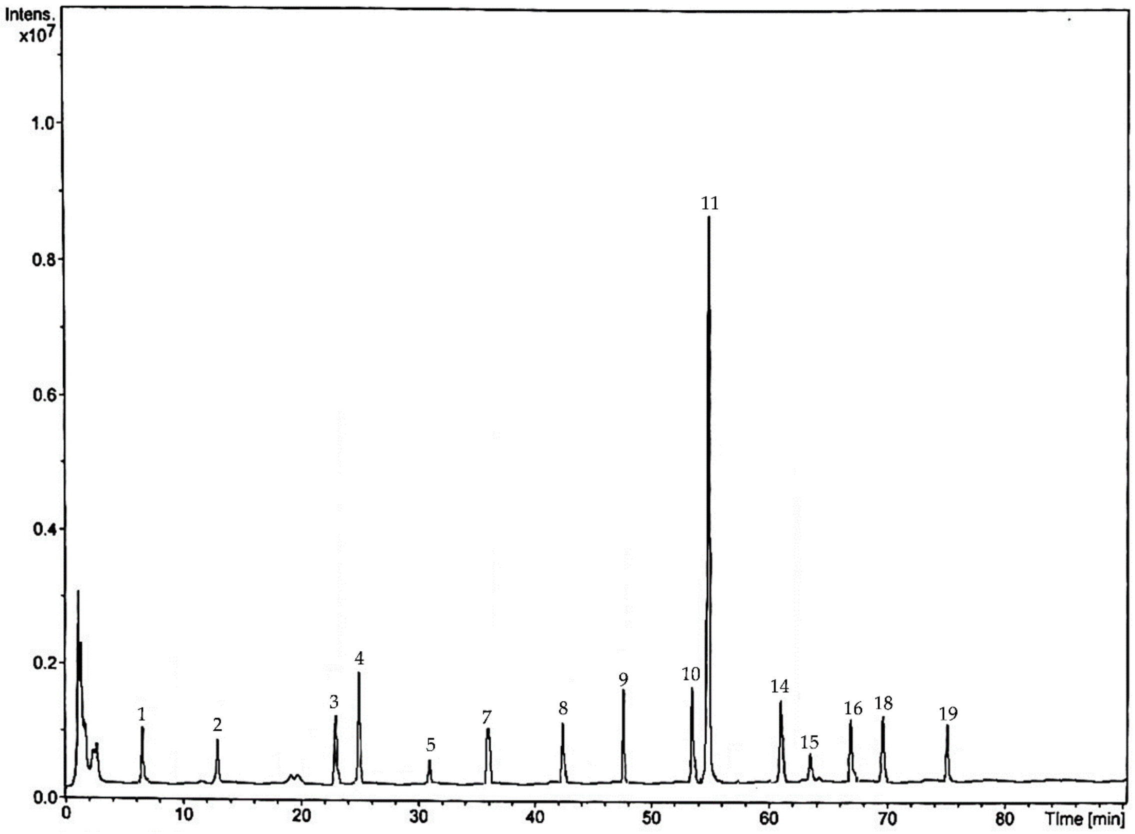

Figure S3. Total ion current chromatogram of USO 31 hempseed meal (HSM). Peak numbers refer to polyphenols reported in Table 3.

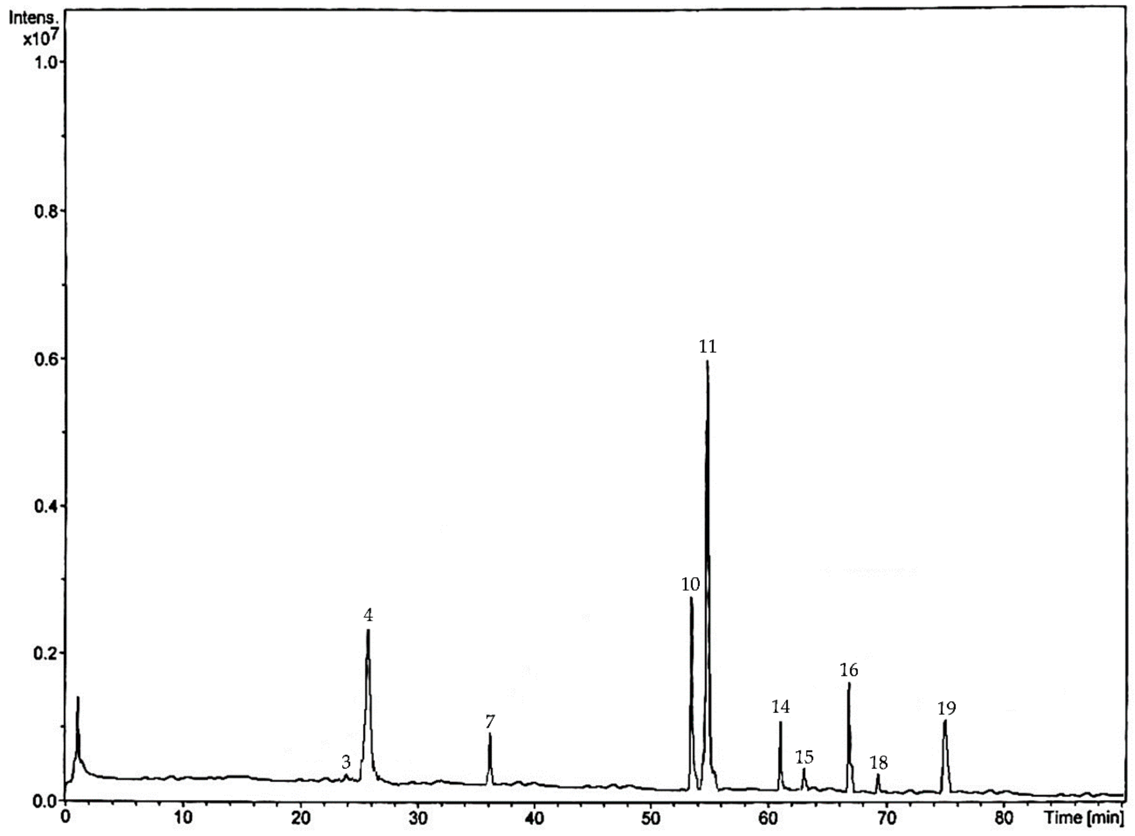

Figure S4. Total ion current chromatogram of Futura 75 hempseed meal (HSM). Peak numbers refer to polyphenols reported in Table 3.
